# Fruits of Black Chokeberry *Aronia melanocarpa* in the Prevention of Chronic Diseases

**DOI:** 10.3390/molecules22060944

**Published:** 2017-06-07

**Authors:** Tunde Jurikova, Jiri Mlcek, Sona Skrovankova, Daniela Sumczynski, Jiri Sochor, Irena Hlavacova, Lukas Snopek, Jana Orsavova

**Affiliations:** 1 Institute for teacher training, Faculty of Central European Studies, Constantine the Philosopher University in Nitra, Drazovska 4, Nitra SK-949 74, Slovakia; tjurikova@ukf.sk; 2 Department of Food Analysis and Chemistry, Faculty of Technology, Tomas Bata University in Zlín, nám. T. G. Masaryka 5555, CZ-760 01 Zlín, Czech Republic; skrovankova@ft.utb.cz (S.S.); sumczynski@ft.utb.cz, (D.S.); ihlavacova@ft.utb.cz (I.H.); lsnopek@ft.utb.cz (L.S.); 3 Department of Viticulture and Enology, Faculty of Horticulture, Mendel University in Brno, Valticka 337, CZ-691 44 Lednice, Czech Republic; sochor.jirik@seznam.cz; 4 Language Centre, Faculty of Humanities, Tomas Bata University in Zlín, nám. T. G. Masaryka 5555, CZ-760 01 Zlín, Czech Republic; orsavova@fhs.utb.cz

**Keywords:** black chokeberry, *Aronia melanocarpa*, bioactive compounds, chronic diseases, prevention

## Abstract

In recent years, growing attention has been focused on the utilization of natural sources of antioxidants in the prevention of chronic diseases. Black chokeberry (*Aronia melanocarpa*) represents a lesser known fruit species utilized mainly as juices, purees, jams, jellies and wine, as important food colorants or nutritional supplements. The fruit is valued as a great source of antioxidants, especially polyphenols, such as phenolic acids (neochlorogenic and chlorogenic acids) and flavonoids (anthocyanins, proanthocyanidins, flavanols and flavonols), particularly cyanidin-3-galactoside and cyanidin-3-arabinoside, as well as (−)-epicatechin units. The berries of *A. melanocarpa*, due to the presence and the high content of these bioactive components, exhibit a wide range of positive effects, such as strong antioxidant activity and potential medicinal and therapeutic benefits (gastroprotective, hepatoprotective, antiproliferative or anti-inflammatory activities). They could be also contributory toward the prevention of chronic diseases including metabolic disorders, diabetes and cardiovascular diseases, because of supportive impacts on lipid profiles, fasting plasma glucose and blood pressure levels.

## 1. Introduction

In recent years, the utilization of natural products has substantially increased following the trends of chronic diseases reduction. The small blueberry-sized black or dark violet color fruits of *Aronia melanocarpa* shrub are of great interest to researchers also because they have, similarly to other distinguished fruit types [1,2,3,4,5,6], one of the highest levels of antioxidants [7]. Berry crops, especially those underutilized (elderberry, honeyberry and black chokeberry), are widely recognized as valuable source of bioactive compounds used as natural food colorants and for health-promoting activities [8]. Our attention has been focused on chokeberries involved in numerous clinical studies due to their antioxidant properties related to the high phenolic content [9,10,11,12,13], especially anthocyanins [14,15] in the form of cyanidin derivatives [16]. It should be emphasized, as well, that other than polyphenolic compounds, the berries are also rich in bioactive constituents, such as vitamins (vitamin C and vitamin E), mineral elements (potassium, calcium and magnesium), carotenoids, pectins, organic acids and carbohydrates present in smaller amounts [17]. That is a reason why *Aronia* species are at the top of fruits and berry species and why they display the wide range of activities in relation to chronic diseases, especially heart and cardiovascular [18,19,20] ones, involving antiatherosclerotic, hypotensive and antiplatelet properties [17]. Due to the presence of these bioactive compounds, the berries of *Aronia* exhibit also other biological, health-promoting effects both in vitro and in vivo, such as gastroprotective (stomach ulcers), hepatoprotective, antiproliferative activities (e.g., colon cancer), prevention and treatment of diabetes [21,22,23,24,25].

*Aronia* fruits, with the common name chokeberry, belong to the *Aronia* genus of the *Rosaceae* family, *Maloideae* subfamily. The genus is represented by two species utilized as fruits: *Aronia melanocarpa (Michx.) Ell*. (black chokeberry) and *Aronia arbutifolia* (L.) *Pers*. (red chokeberry). Both species are considered as noted ornamental landscape shrubs. Black chokeberry is nowadays a widely utilized crop for the production of natural food colorants [26] and due to its usage in processed products [27] and nutraceutical utilization in the form of nutritional supplements [28].

Black chokeberry originates from the eastern parts of North America. The fruits of *A. melanocarpa* have been applied already by Native Americans to cure colds. The center of *Aronia melanocarpa* is located in the northeastern states of the USA and the Great Lakes region, with a range extension into the higher altitude of the Appalachian Mountains. In the United States, there are primary cultivars available for the fruit production: ‘Viking’ and ‘Nero’ cultivars. In comparison to wild-type chokeberry fruits, the commercial cultivars are larger, sweeter and have a bigger production [29].

The cultivation of this crop for the food industry in Europe started in Russia in the early 1900s in the cold areas of Siberia, and afterwards, the plant was spread all over Russia. In the first half of the 20th century, the *Aronia* plant was introduced also to other European countries, such as Eastern European states (e.g., Poland with a current area around 1600 ha and a production of 14,000–15,000 t), Germany, Finland, Sweden and Norway [30]. To the significant commercial black chokeberry cultivars besides ‘Viking’ (Finland) and ‘Nero’ (Czech Republic) belong some other cultivar types such as ‘Aron’ (Denmark), Polish ‘Galicjanka’, Swedish ‘Hugin’, Russian ‘Rubina’ or ‘Fertödi’ (Hungary).

*Aronia* fruits can ripen as early as mid-July, but they primarily ripen during the month of August [31]. For the optimal harvest date, regarding the maximum reached levels of berry weight and anthocyanin content, choosing the early September days could be the most suitable [29].

The taste of mature black chokeberry fruits is sweet with the level of reduced sugars in chokeberry cultivars from 8% (‘Viking and Nero’) to about 12% (‘Hugin’) [32]. For these berries, it is typical also a certain level of astringency, due to the tannin presence, so they are not so popular for eating as natural fresh fruits. At present, black chokeberries are therefore used mainly as ingredients in the processed form, separately or more often together with other fruits, to make juices [33], purees [34], jams, jellies, wine [35], syrups, teas, alcoholic or energizing drinks and flavorings for products, such as yogurts [36]. Furthermore, by-products such as pomace are a great source of bioactive constituents, comparable to berries [37]. The berries can be also utilized as food colorants [17,38]. For this purpose, solid-phase extraction (SPE) [39], supercritical fluid extraction (SFE) or ultrasound-assisted extraction (UAE) [40,41,42] are highly recommended.

This review aims to summarize particular phenolic components of the lesser known fruits of black chokeberry (*Aronia melanocarpa*), their bioavailability, antioxidative properties and health-promoting benefits in relation to chronic diseases. Also shown are the new trends of fruit utilization and its situation among other valuable sources of berry crop and lesser known fruit species.

## 2. Polyphenols as the Main Bioactive Compounds of Chokeberry Fruit

Nowadays, the *A. melanocarpa* fruit is valued as a great source of bioactive components, such as polyphenols. They are considered to be very important dietary antioxidants [43,44].

Antioxidants are the substances that can scavenge free radicals, instable and reactive forms with an unpaired electron in the outer orbit. Free radicals could cause cell damage to the human body, “oxidative stress” that leads to various chronic diseases, such as atherosclerosis, inflammation, cancer and neurodegenerative diseases. Some dietary antioxidants are capable of donating hydrogen radicals and, thus, may act as free radical scavengers. Therefore, these compounds may prevent some crucial points in disorders’ progress.

First of all, black chokeberry fruit has a high content of total polyphenol (TP). Total polyphenols in *Aronia melanocarpa* were determined in the range of 690–2560 mg gallic acid equivalents (GAE) in 100 g fw (fresh weight) [44,45,46,47]. 

It is also important to emphasize the remarkable position of black chokeberry fruit among other berry crops. The total polyphenols (TP) in *A. melanocarpa* berries were quantified as higher than for many other remarkable berries, including blueberries, red raspberries, red currant, strawberries and blackberries [44,46,48]. The comparison of TP content in various important fruit berries confirms that the amount of TP in *A. melanocarpa* berries is similar to black currant (*Ribes nigrum*) content, about 2–4-fold of the content in blackberry (*Rubus fruticosus*), 4-fold for blueberry (*Vaccinium corymbosum*), 3–8-fold for red raspberry (*Rubus idaeus*) and 10-times more than in strawberry (*Fragaria ananassa*). Red currants (*Ribes rubrum*) have about 2/3 of the black chokeberry TP content; lingonberry (*Vaccinium vitis-idaea*) has about 1/4; and cranberry (*Vaccinium macrocarpon*) approximately 1/8 of the *A. melanocarpa* fruit phenolics [43,45,46,47]. Tian et al. [49] compared and analyzed the TP content of 13 various berry crops by the HPLC method with DAD detection. The results of the experiment showed that the highest concentration of polyphenols was present in bilberry fruit (564 mg/100 g fw); the rest of the berry crops had a content of TP similar to black chokeberry (432 mg/100 g), lingonberry and hawthorn (440 mg/100 g).

To achieve a maximum yield of polyphenols in black chokeberry fruit, some factors must be taken into account. The content of total polyphenols (TP) and particular phenolic compounds of different types of berries depend on many factors, such as genetic attributes (cultivar, genotype, variety) or growing conditions (location, cultivation techniques, ripening stage), processing and storage [43,45,50,51]. The optimum for storage is a temperature of 3 °C, although after six months of storage at 3 °C, the content of total polyphenols decreases only by 30% [52]. High variations and discrepancy in total polyphenolic content during different growing seasons are due to the different air temperatures, sunlight and rainfall intensity, as has been proven by Tolic et al. [9]. Higher temperatures and bright sunshine hours caused the high content of TP correlated with anthocyanins.

To obtain a maximum yield of polyphenols, a fruit cultivar must be taken into account as a very important factor [45,47,53]. The cultivars such as ´Viking´ and ´Nero´ and even wild varieties have higher amounts of TP than cultivar ´Galicianka´ [43]. Cultivar ´Hugist´ represents the best source of polyphenols (23.4 mg of gallic acid equivalents per g of fresh fruit weight) as has been proven by Ochmian et al. [32]. Otherwise, the lowest value of TP was detected in ´Aron´ cultivar (15.86 mg of gallic acid equivalent per fw) [54]. It should be considered that in the case of the determination of total polyphenols, different methods and conditions of plant growing must be taken into account as well. Moreover, the extraction process is important for the optimal results of polyphenol content and composition. Various solvents (type, concentration), particle size, solid–solvent ratio, pH and extraction time could have an impact. Among these variables, time was ascertained as not a statistically important factor for the extraction of polyphenols. As the optimal extraction conditions, maceration of 0.75 mm-sized berries by 50% ethanol, with a solid–solvent ratio of 1:20, was evaluated [55]. Furthermore, the integrated extraction-adsorption process, simultaneous phenolics extraction and their purification in a single operation could be used for the enhancement of the extraction yields of phenolics and enrichment of the *Aronia* extracts in antioxidant phenolics up to 15 times [56].

The polyphenols content and polyphenolic profile of *Aronia melanocarpa* berries and different types of products, such as pomace or processed juice, were determined by several researchers [7,53]. The pomace has been identified as the product with the highest content of phenolics in comparison to juice or fruits. The average concentration of polyphenols in pomace was about five-fold higher compared to chokeberry juice. 

The effect of different drying methods, such as freeze-drying, vacuum, convective drying and the microwave method, on the quality factors of chokeberry fruit was studied Samoticha et al. [57]. The results showed that the quality of dried chokeberry depends on the method and conditions of fruit drying. The highest content of bioactive compounds they determined was in freeze-dried samples, compared with fresh fruits. The increase in air temperature during drying deteriorated dried product quality due to the content of phenolics. 

A phenolic analysis of dried chokeberry, bilberry and black currant fruit teas, prepared by decoction and infusion, showed that the highest concentration of phenolics is in chokeberry teas, followed by bilberry and black currant teas [58]. Optimization of the extraction process for maximal yield of total phenolics content by sonication was determined by Ramić et al. [59]. Ultrasonic power, temperature (30–70 °C) and extraction time (30–90 min) were investigated. The most suitable conditions were evaluated as a power of about 200 W, temperature of 70 °C and 80 min of extraction.

To the most important polyphenols of black chokeberries belong phenolic acids and flavonoids such as anthocyanins, flavanols, flavonols and proanthocyanidins [43]. As the most significant phenolic compounds were identified from the group of phenolic acids hydroxycinnamic acids, especially neochlorogenic acid, from anthocyanins, there were cyanidin-3-galactoside and cyanidin-3-arabinoside; from proanthocyanidins, procyanidin B1. Flavanols (epicatechin) and flavonols (mainly quercetin glycosides) are minor components of black chokeberry fruits [43,48].

### 2.1. Phenolic Acids of Chokeberry Fruit

Generally, berries are a rich source of hydroxycinnamic acids, derivatives of slightly water-soluble cinnamic acid. The most abundant is chlorogenic acid, which is a complex of caffeic acid linked to quinic acid through an ester bond. Together with neochlorogenic acid [60], they are considered to be the major non-flavonoid polyphenolic compounds in chokeberries [3,61]. Rop et al. [45] confirmed the fact that chlorogenic and neochlorogenic acids are very important antioxidants by the experiments of five black chokeberry cultivars ´Aron´, ´Fertödi´, ´Hugin´, ´Nero´ and ´Viking´. Furthermore, Ochmian et al. [32] determined chlorogenic and neochlorogenic acids as significant phenolic acids in similar amounts in cultivars ´Hugin´, ´Nero´, ´Viking´ and ´Galicianka´. The content of chlorogenic acid, assessed by Jakobek et al. [43], was quantified as a little bit higher. Zheng and Wang [47] drew attention to caffeic acid and its derivative present in wild chokeberry and their importance on the total antioxidant capacity. The significant amount of caffeic acid and also ferulic acid was detected by Hakkinen et al. [62]. The content of total phenolic acids is comparable to Saskatoon berries. The predominant phenolic acids are present as 3-*O*-caffeoylquinic acid, which makes up about 23% of the total phenolic content, and 5-*O*-caffeoylquinic acid, with about 11% [49]. During the process of chokeberry juice pasteurization (80 °C), the most unstable among the phenolics were hydroxycinnamic acids with losses up to 59%. On the contrary, plasma-treated chokeberry juice showed an increased concentration of hydroxycinnamic acids by 23% [63].

### 2.2. Flavonoids of Chokeberry Fruit

The main flavonoid subgroups of black chokeberry fruits are represented by anthocyanins, proanthocyanins, flavonols and flavanols (catechins) [45,53]. Flavonol glycosides represent about 10% of total phenolics content with the predominance of quercetin 3-*O*-galactoside (Q-Gal) and quercetin 3-*O*-glucoside [49]. Similarly to the content of total phenol, also flavonoid and proanthocyanidin contents of black chokeberry extracts are higher than those of other berry extracts such as blueberries, black currant, blackberry, bilberry, red currant, red raspberry and strawberry [44,46,48].

#### 2.2.1. Anthocyanins

Much of the interest in *Aronia melanocarpa* fruit is focused on anthocyanins, glycosylated pigmented flavonoid compounds, and their content. Among all detected compounds in black chokeberry fruit, anthocyanins were established as very important compounds [64], sometimes dominating ones [44]. Due to Oszmiański and Wojdylo [53], anthocyanins represent about 25% of all phenolic components; according to Jakobek et al. [44], this is up to 41%. The major anthocyanin of black, purple and red *Aronia* (*Aronia melanocarpa*, *Aronia prunifolia*, and *Aronia arbutifolia*) was identified as cyanidin-3-galactoside [65]. Ochmian et al. [32,61] determined more than 50% of anthocyanins of the present polyphenols when even one concrete compound (cyanidin-3-*O*-galactoside) made up 50% of the determined phenolic compounds in four studied cultivars.

Semi-preparative HPLC, thin-layer chromatography and spectral techniques indicated cyanidin as a single aglycone and glucose, galactose, arabinose and xylose as associated sugars. The anthocyanin composition is represented almost exclusively by cyanidin glycosides, namely predominant compounds cyanidin-3-*O*-galactoside and cyanidin-3-*O*-arabinoside, further cyanidin-3-*O*-glucoside and cyanidin-3-*O*-xyloside [43,48,50,66]. Furthermore, in chokeberry teas (infusion and decoction), the main components are cyanidin-galactoside and cyanidin-arabinoside, while cyanidin-glucoside and cyanidin-xyloside are present in half the amount. The content of cyanidin glycosides in natural chokeberry juices is similar as in chokeberry decoctions [58]. The major cyanidins were cyanidin-3-*O*-galactoside (222 mg/100 g) and 3-*O*-arabinoside (Cy-Ara, 159 mg/100 g) [49]. Slimestad et al. [67] reported total anthocyanins content in chokeberries (481 mg/100 g fw after extraction by 0.1% hydrochloric acid in methanol) with the major anthocyanidins cyanidin 3-*O*-galactoside (65% of total anthocyanins) and cyanidin 3-*O*-arabinoside (30%). Compared to anthocyanin aglycons, cyanidin shows higher antioxidant activity with the order of antioxidant potency values: cyanidin > delphinidin > malvidin ≈ peonidin ≈ petunidin [47].

Numerous studies focused attention on the comparison of anthocyanins content in different types of berry crops. For example, among berry fruits, such as black currant (*Ribes nigrum*), red currant, (*Ribes rubrum*), gooseberries (*Ribes grossularia*), strawberry (*Fragaria ananassa*), blackberry (*Rubus fruticosus*) and red raspberry (*Rubus idaeus*), black chokeberry has the highest total anthocyanin concentration [44,46,66]. According to the studies of Baloghova et al. [68], there are no significant differences in anthocyanin content between fruits of black chokeberry and blueberry (*Vaccinium corymbosum)*. The content of anthocyanins in chokeberry is also comparable to the elderberry (*Sambucus nigra*) variety ´Haschberg´; cultivar ’Rubini’ has a higher amount of anthocyanins [69].

The content of anthocyanins is species dependent; their highest content was confirmed in black chokeberry and the lowest in red chokeberry fruits [65]. The black chokeberry anthocyanin content depends on cultivar, as well; higher total anthocyanin content was determined in cultivars “Viking” and “Nero”, similarly to the higher polyphenol content, compared to the wild type of black chokeberry [43].

Quite interesting findings of total anthocyanins content are about their amount in the fruit decoctions and infusions. Regarding three types of the fruits containing higher concentrations of anthocyanin colorants (chokeberry, bilberry, black currant), the highest amount was determined by Šavikin et al. [58] in the bilberry decoction and infusion. In chokeberry and black currant teas, there was a two-fold lower anthocyanin content than in bilberry samples. Compared with the content in dry fruits, 1–3% of total anthocyanins were extracted into teas.

Another important task in relation to berries’ utilization is the stability of anthocyanins. 

First of all, the total yield of anthocyanins is significantly influenced by temperature. Wilkes et al. [15] examined the stability of anthocyanins during juice processing within six months of storage at 25 °C. Anthocyanins were extensively degraded by thermal treatment, and their concentration declined linearly. The lower storage temperature together with the pH value of black chokeberry juice led to the highest yield of anthocyanins in juice, as has been proven in the experiments of Howard et al. [26].

Secondly, the anthocyanin content in fruit is influenced by the extraction procedure. It has been proven that the most suitable procedure to obtain the highest yield of anthocyanins is the usage of acidified methanol as the extraction solvent [4]. Park and Hong [70] investigated the content of anthocyanins of *Aronia melanocarpa* extracts depending on extraction solvents, such as hot water, 50% ethanol and 50% methanol. The total anthocyanin content of the extract yield was higher with 50% methanol solvent than with the usage of 50% ethanol or hot water as the medium. In order to isolate major anthocyanins from *Aronia* berries, an extraction with methanol containing trifluoroacetic acid could be performed, since direct alcoholic extractions provide very poor yield, not keeping anthocyanins in the stable flavylium cationic form [71]. One of the most efficient extractants of anthocyanins is supposedly Na_2_SO_3_. The presence of a preserve sodium sulfate (IV) in *Aronia* solutions in the filtration process (nanofiltration) increases the efficiency of the process [72].

Furthermore, the investigation of the temperature effect in combination with other extraction conditions is important and interesting. The influence of temperature (20–70 °C), extraction time (0–240 min), solvent composition (0–50% ethanol in water) and ultrasound power (0–100 W) on anthocyanins yields was studied by Zeković et al. [40] and D’Alessandro et al. [41]. Results showed that higher temperature and ethanol content in the solvent greatly improved the extraction yields. Ultrasound improved mainly the extraction kinetics. The ultrasound effect was higher in the beginning of the extraction process and at low temperatures.

In the study of Yong et al. [73], the influence of various storage conditions (different temperatures) in combination with different forms of extract (powder or solution) on extract stability was examined. In the solution, a decrease was detected after four weeks of storage under the same conditions. The storage over 40 °C caused the decrease of cyanidin-3-*O*-galactoside in both evaluated forms. The optimum temperature for storage, with respect to no changes in anthocyanins, was established at 4 °C. 

In another study [8], the influence of blanching, freezing, maceration temperatures (2–50 °C) and enzyme treatment before juice pressing on anthocyanin content was examined. The results of the experiment showed that the best results were achieved by cold maceration of frozen berries without enzyme addition to the pomace. On the contrary, cold and hot maceration of fresh, not blanched, berries with enzyme gave the lowest yield of anthocyanins.

Kovačević et al. [63] monitored changes in anthocyanins subjected to the effect of cold atmospheric gas phase plasma. The results were compared with the control sample (untreated) and pasteurized chokeberry juice. The treatment of cold atmospheric gas phase plasma exhibited 23% loss of anthocyanins in comparison to untreated juice. 

#### 2.2.2. Flavonols and Flavanols, Proanthocyanins

Flavonols (quercetin glycosides) and flavanols (flavan-3-ols, epicatechin in proanthocyanidins) are also present in the berries, but only as minor components. 

In the chokeberry fruit, a mixture of four different flavonols is present, mainly quercetin-3-galactoside (hyperoside), quercetin-3-glucoside (isoquercetin), quercetin-3-rutinoside (rutin) [32,44,74,75] and quercetin-3-robinobioside [67]. Furthermore, quercetin 3-vicianoside was found in *A. melanocarpa* fruit, though in the lowest concentration [32]. Further, another flavonol identified in *Aronia*, kaempferol, is present only in a much smaller amount [44].

In the chokeberry decoctions and infusions, rutin was assessed as the predominant compound, followed by hyperoside (half amount) and isoquercitrin (one third amount) [58]. Generally, quercetin aglycons have been found in a wide range of lesser known fruit berries, such as bog whortleberry and lingonberry, where the contents were the highest, lower in cranberry, sweet rowan, rowanberry, sea buckthorn berry and crowberry [76]. During the process of chokeberry juice pasteurization, the content of flavonols was increased by 5%, as has been reported by the study of Kovačević et al. [63].

Polymeric procyanidins, the chains of flavan-3-ol subunits, are a significant group of polyphenols in chokeberry. They are composed predominantly of (−)-epicatechin units. The units are connected mainly with C4-C6 and C4-C8 bonds (B-type bonds). Free epicatechin is also present in black chokeberries, although its concentration is significantly lower in comparison with polymeric procyanidins (catechins). The degree of polymerization of procyanidins varies from 2–23 units in the fruits. The degree of polymerization is important for their bioavailability as low molecular weight oligomeric procyanidins are absorbed intact in the gastrointestinal tract, but polymerization greatly impairs intestinal absorption [71,77].

Similar oligomeric proanthocyanidins were identified in wild and cultivated *A. melanocarpa* fruits; however, the higher flavonoid content was determined in the berries of wild *Aronia*. In genotypes of *Aronia*, the presence of about 64 different isomers, such as proanthocyanidin dimers consisting of catechin and/or epicatechin, depending on the structural stereochemistry and configuration, was identified [78].

Procyanidins in the chokeberries are present in the flesh by about 70%, 25% in the skin and 5% in the kernels [37]. In bark and berries of the *Aronia* plant, there are important dimeric procyanidins B2 and B5 and trimeric procyanidin C1 [71].

Three *A. melanocarpa* cultivars ‘Moskva’, ‘Hugin’ and ‘Nero’ and also hybrid *A. prunifolia* were analyzed to determine the procyanidin concentration. The highest amount was found in *A. prunifolia*, slightly less in the ‘Nero’ cultivar and the least in the cultivar ‘Hugin’ and ‘Moskva’, with nearly half the content of procyanidin compared to *A. prunifolia* [50]. Black, purple and red *Aronia* (*Aronia melanocarpa*, *Aronia prunifolia* and *Aronia arbutifolia*) and ‘Viking’ (*Aronia mitschurinii*) berries were quantified for procyanidin content. ‘Viking’ *Aronia* berries and the red variety had substantially more proanthocyanidins than the other fruits [65].

As Mayer-Miebach et al. [37] detected, there are no significant changes regarding procyanidins content during the commercial juice production. Squeezing increases the procyanidins content of the mash by about 30% as compared to the berries, maybe due to an enhanced extractability and subsequent release of monomeric units (predominantly epicatechin) and of oligo- and poly-meric procyanidins. Heating of mash or raw juice did not affect procyanidins; the content remained stable after heating chokeberry purees up to 100 °C within 20 min and holding for 15 min. A similar concentration of total procyanidins was determined for the raw juice, the decanted juice and the sterilized juice. The procyanidins content in the sterilized juice and in the berries was similar, but significantly lower than in the heated mash (about 20%). The procyanidins content in the pomace was significantly higher than in the berries (about 45%) and also higher than in the heated mash (about 10%).

#### 2.2.3. Bioavailability of Black Chokeberry Polyphenols

The bioavailability of chokeberry components is discussed because of the efficiency of particular phenolic components in the organism. The antioxidants should be absorbed, transported, distributed and retained properly in the biological fluids, cells and tissues. Generally, the bioavailability of chokeberry phenolics seems to be quite low [79].

The metabolism of black chokeberry anthocyanins has been mostly studied in rat models. Despite the predominate position of anthocyanins among polyphenols, their absorption is very poor and low for the small intestine (in the case of cyanidin 3-glucoside 22.4%, cyanidin 3-galactoside 13.6%) and stomach, as well [80,81,82]. After absorption, anthocyanins are transported with the circular system to various organs and tissues, including the liver, heart, prostate, testes, brain and body fat, but they are mostly distributed to the urinary bladder and kidney of rats [83,84]. The low rate of absorption of anthocyanins and another polyphenolic compounds in black chokeberry fruit can be explained by their transformation, modification by methylation or conjugation with glucuronic acid [81,85,86]. For example, cyanidin 3-glucoside is methylated and glucuronidated, cyanidin 3-galactoside oxidized, and finally, all derivates (conjugates) are eliminated by the urinary system [82,86,87]. Another way is an extraction with the bile [81] or with the feces [85]. On the other hand, anthocyanin conjugation is probably responsible for their metabolic activation and health benefits in relation to chronic diseases [17,87].

Other studies are focused on the metabolism of phenolic acids. Raimondi et al. [88] found that *Bifidobacterium animalis* is able to hydrolyze chlorogenic acid into caffeic acid by the presence of the intracellular enzyme feruloyl esterase. Chlorogenic acid is already metabolized in the digestive system thanks to colonic microflora to quinic acid and caffeic acid [89], which are then absorbed in the small intestine [90] and transported into the blood circulation [91]. Metabolites of chlorogenic acid (dimethoxycinnamic acid, glucuronide derivatives and sulfated caffeoylquinic acid derivatives) are excreted mainly with the urine [17,92].

## 3. Antioxidant Activity of Chokeberry Fruit

The determination of antioxidant activity is one of the ways to define the protection ability against free radicals. It is characterized as the capability of the compound (or mixture of compounds) to inhibit the oxidative reaction of various biomolecules [93]. In recent years, *A. melanocarpa* has been highlighted with the respect to its particularly great quantity of antioxidants. *Aronia* antioxidants are represented mainly by vitamin C and polyphenols, such as anthocyanins, phenolic acids, flavanols, flavonols and tannins [44,46,53]. 

The antioxidant effects of chokeberry extract could be evaluated by different in vitro assays, such as radical-scavenging activity by the DPPH test with 2,2-diphenyl-1-picrylhydrazyl, the TEAC method (Trolox-equivalent antioxidant capacity) and the ABTS radical (2,2′-azino-bis(3-ethylbenzothiazoline-6-sulfonic acid)) or FRAP (ferric reducing antioxidant power) assay with the reduction of complexes of 2,4,6-tripyridyl-s-triazine (TPTZ) with ferric chloride hexahydrate (FeCl_3_·6H_2_O) [93].

In vitro investigations show an increase in antioxidant defense and a decrease in reactive oxygen species levels after incubation with chokeberry bioactive compounds [45,94,95]. Polyphenol compounds derived from the *Aronia* berries have been investigated and detected to have a distinct effect on the reduction of the plasma lipid peroxidation induced by ziprasidone in vitro [96].

In vivo studies of chokeberry bioactive compounds’ effect are limited. Chokeberry anthocyanins could decrease lipid peroxidation, as was shown in the animal model [25]. The mechanisms of the in vivo antioxidant activity of phenolics after absorption spread out far beyond radical scavenging and include suppressing the formation of reactive oxygen species and reactive nitrogen species, inhibition of prooxidants and restoration of antioxidant enzymes, as well as probably cellular signaling to regulate the level of antioxidant compounds and enzymes [96].

Oszmiański and Wojdylo [53] determined the antioxidant activity of *Aronia melanocarpa* berries and their products such as pomace and processed juice. The results showed that the pomace has a much higher antioxidant activity than berries or juice. This fact is in accordance with the highest measured content of phenolics in pomace. Predominant polyphenols consisted of polymeric proanthocyanins together with (−)-epicatechin. The concentration of phenolic acids (chlorogenic and neochlorogenic acids) in juice was higher than in pomace. The comparative study of Rop et al. [45] confirmed the highest antioxidant activity in the same type of black chokeberry fruit product, pomace, followed by *Aronia* fresh berry fruits and juices.

The highest antioxidant activity of dried *Aronia* berries, with flavonoids as the predominant compounds, was reported by Tolić et al. [97]. Dried berries were followed by fruit tea (dried and ground chokeberry pomace), powder samples from pulp, fruit and pomace, further capsules (chokeberry extract) that had about twice lower values of the antioxidant activity and juices with even seven-times less values compared to dried berries. Anthocyanins represented a significant fraction of total phenolics in powder and capsule samples.

The antioxidant activity values, similarly to the polyphenols content, are species dependent. The hybrid of *A. prunifolia* was found to have the highest antioxidant activity and to be the richest in polyphenols, proanthocyanidins and anthocyanins compared with *A. melanocarpa* cultivars ´Viking´ and ´Aron´ [98]. In the case of the scavenging effect of *A. melanocarpa* cultivars ´Aron´, ´Fertodi´, ´Viking´, ´Hugin´ and ´Nero´ on reactive oxygen species, the significant variability of black chokeberry fruit methanolic extracts was evident [45].

In comparison with other well-known and lesser known fruit species, relatively higher values of antioxidant capacity were reported for chokeberry fruit [44,45,47]. Zheng and Wang [47] compared the antioxidant activity of wild chokeberry, blueberry (*Vaccinium corymbosum*), cranberry (*Vaccinium macrocarpon*) and lingonberry (*Vaccinium vitis-idaea*) fruit. The chokeberry had significantly higher antioxidant activity related to the higher content of anthocyanins and phenolics than the other three berries. The contribution of individual phenolics to the total antioxidant capacity was generally dependent on their structure and content in the berries. Phenolics such as quercetin and cyanidin, with 3′,4′-dihydroxy substituents in the B ring and conjugation between the A and B rings, had highly effective radical scavenging structures in the berries. Phenolic acids, such as caffeic acid, displayed high antioxidant activity, as well, probably due to their dihydroxylation in the 3,4 positions as hydrogen donors.

The antioxidant activity of *A. melanocarpa* fruit is mostly contributed by the polyphenolic compounds [4]. Various types of phenolic compounds may contribute differently to the total antioxidant activity. Rugină et al. [98] found a positive correlation between antioxidant activity and total proanthocyanidin and anthocyanin content in berries of *Aronia melanocarpa* cultivars ´Viking´, ´Aron´ and hybrid *Aronia prunifolia*. In the case of cultivars ´Nero´, ´Aron´ and ´Fertodi´ cultivated in the Czech Republic [45], a higher correlation in the case of neochlorogenic acid than of chlorogenic acid was found. The correlations between antioxidant activity and cyanidin 3-arabinoside and (−)-epicatechin were even higher followed by cyanidin 3-galactoside.

The lipophilic and hydrophilic antioxidant capacities, measured by oxygen radical absorbance capacity in berries of black chokeberry, *Ribes nigrum* (black currant), *Ribes rubrum* (red currant), *Ribes grossularia* (gooseberries) and *Sambucus nigra* (elderberry), were provided by Wu [65]. However, the lipophilic antioxidant capacity was quite low; the hydrophilic capacities for black currant, chokeberry and elderberry are among the highest of fresh berry fruits. The best linear relationship was observed with total phenolics, followed by total anthocyanins and proanthocyanidins. 

## 4. Chokeberry Fruit and Its Health-Promoting Activity

The berries of *A. melanocarpa* belong to the group of fruits with one of the highest in vitro antioxidant activities due to the presence and high content of bioactive components, mainly polyphenols (phenolic acids, flavonoids, such as anthocyanins, proanthocyanidins, flavanols and flavonols). The high antioxidant activity of berries makes their effective utilization in the treatment of chronic diseases related to oxidative stress possible, especially diabetes, cardiovascular diseases and cancer. Except for the mentioned properties, numerous other positive medicinal and therapeutic benefits, namely immunomodulatory, antibacterial, hepatoprotective, gastroprotective and anti-inflammatory, have been demonstrated for black chokeberry extracts both in vitro, in cells or cell lines, and in in vivo studies in humans or animals [22,23,24,99,100,101,102,103]. 

### 4.1. Anti-Inflammatory Effect of Chokeberry Fruit as a Base in the Prevention of Chronic Diseases 

The anti-inflammatory properties of black chokeberry fruit are related to the prevention of the development of chronic diseases, such as diabetes [20], cardiovascular diseases [102] and chronic problems with the immune system [104]. 

Nowadays, the fruit of black chokeberry is highlighted in relation to strengthening the human immune system. It can be explored by different mechanisms of action, such as inhibition of release of cytokine IL-6, IL-8 and TNF-α in the human monocytes and activation of NF-κB and prostaglandin E_2_ (PGE_2_) [104]. 

Berries of black chokeberry possess an immunomodulation effect. The complement-modulating activities, the inhibitory activities on nitric oxide production in LPS-induced RAW 264.7 mouse macrophages of procyanidins C1, B5 and B2 and anthocyanins of *Aronia melanocarpa* were examined by Ho et al. [101]. Results of the experiment showed that procyanidins C1, B5 and B2 and anthocyanins are mainly responsible for the immunomodulation effect. Cyanidin 3-glucoside possessed stronger activity than the other anthocyanidins. Xu and Mojsoska [102] assessed the immunomodulation properties in lipopolysaccharide (LPS)-stimulated human monocytes mono mac 6. The isolated chokeberries anthocyanin fraction of six different anthocyanidins exhibited strong antioxidative capacity. However the immunomodulatory activity is not associated with *Aronia* anthocyanins; they had only a slight effect on reducing IL-10. Therefore, there are other *Aronia* bioactive compounds responsible for that attribute.

Martin et al. [105] characterized the anti-inflammatory effects of *Aronia* berries and their polyphenols using primary C57/BL6 mouse splenocytes. The berries of commercial *Aronia* cultivar 'Viking' and extracts inhibited LPS-stimulated IL-6. *Aronia* extracts inhibited IL-6 predominately in CD4-lymphocytes. Cyanidin 3-arabinoside and quercetin inhibited LPS-stimulated IL-6; moreover, quercetin also inhibited LPS-stimulated IL-10.

#### Anti-Inflammatory Effect of Black Chokeberry Fruit as a Base of Antibacterial and Antiviral Activity

The berries of black chokeberry have antibacterial and antiviral effects, so they can be utilized in the prevention of inflammation. Anti-inflammatory activity in combination with the antimicrobial effect of plant extracts are generally based on phenolics (simple phenols, phenolic acids, quinones, flavones, flavonoids, flavonols, tannins and coumarins), terpenoids and essential oils, alkaloids, lectins and polypeptides [106]. Very effective antimicrobial components are anthocyanins in berry crop [3,107]. The antimicrobial activity of berries and other anthocyanin-containing fruits is likely to be caused by multiple mechanisms and synergies because they contain various compounds including anthocyanins, weak organic acids, phenolic acids and their mixtures of different chemical forms [108]. The investigations of Valcheva-Kuzmanova and Belcheva [24] demonstrated in vitro bacteriostatic activity of *Aronia melanocarpa* fruit juice against *Staphylococcus aureus* and *Escherichia coli*. Liepiņa et al. [109] examined the antimicrobial activity of aqueous and ethanolic extracts from fresh, dried and frozen black chokeberry fruits. The results showed that extracts exhibited antibacterial activity against Gram-positive bacteria *Bacillus cereus* and *Staphylococcus aureus*, without antifungal influence. The extract inhibited also the growth of Gram-negative bacterium *Pseudomonas aeruginosa*, but did not have an influence on *Escherichia coli*. Bräunlich et al. [110] evaluated the ability of *Aronia melanocarpa* extracts and their compounds to prevent biofilm formation and to inhibit bacterial growth of *Escherichia coli* and *Bacillus cereus* in vitro. The ethanolic extract was the most potent inhibitor, compared to dichloromethane and water extracts, of *B. cereus* biofilm formation. As for *Aronia* compounds (flavones, chalcones, flavonols, flavans, flavanones, isoflavonoids, neoflavonoids and dihydroflavonols), they possess anti-biofilm activity without toxicity to the screened species. This non-toxic inhibition may confer a lower potential for resistance development compared to conventional antimicrobials. Black chokeberry juice consumption seems to be effective also in urinary tract infections (UTI) treated by antibiotics, as was shown in the study of Handeland et al. [111]. During a period of six months, the juice (156 mL per day), which was characterized by a high content of total phenolics including B-type procyanidins, anthocyanins and chlorogenic acids, was applied to residents in nursing homes. Urinary tract infection comprised 55% of all medically-treated infections during the study period. The results revealed no immediate reduction in the frequency of UTI or the total use of antibiotics; however, during the period of juice administration, a reduction in antibiotics toward UTI was observed.

A virucidal assay was used to test *Aronia*’s anti-influenza efficacy against different strains of seasonal and oseltamivir-resistant influenza virus in the study of Park et al. [112]. The *Aronia* fruit that contains several polyphenolic constituents possesses in vitro and in vivo efficacy against different subtypes of influenza viruses (H1/K09, H3/PE16, B/BR60), including an oseltamivir-resistant strain (H1/K2785 and HPAI rH5/IS06). At low concentrations, *Aronia* was able to inhibit almost 70% of viral plaques from the H1 and H3 virus, as well as the oseltamivir-resistant strain H1/K2785. These effects were attributed to two constituents, ellagic acid and myricetin, which could be used as influenza therapeutics. Antiviral activity against type A influenza virus was also confirmed by the research of Valcheva-Kuzmanova and Belcheva [24].

### 4.2. Gastroprotective and Antidiabetic Effect of Chokeberry Fruit

It is well known that oxidative stress and inflammatory processes play a key role in the development of diabetes and metabolic syndrome, as well [112,113]. Chokeberry fruits are known also for their gastroprotective effects as many flavonoids are noted for their antioxidant and anti-inflammatory properties [112].

Peptic ulcer is a complex disease that involves infection of the gastric mucosa. The aim of the study of Valcheva-Kuzmanova et al. [22] was to investigate the effect of *A. melanocarpa* fruit juice in a male Wister rat model of indomethacin-induced gastric mucosal damage and its possible interference with the gastric oxidative status. Gastric mucosal concentrations of malondialdehyde (MDA), reduced and oxidized glutathione and blood plasma concentrations of MDA were used as biochemical markers of oxidative stress. The results of the experiment showed that fruit juice reduced indomethacin-induced gastric mucosal injury. The gastroprotective effect of the juice was accompanied by a significant decrease in lipid peroxidation.

Beneficial effects of long-term consumption of chokeberry juice and other *Aronia* products on metabolic parameters including fasting plasma glucose and lipid profiles have also been reported [20,114,115,116]. The berries of black chokeberries effectively improve the glucose metabolism, so they seem to be a good choice in the treatment of diabetes. Chokeberries are a rich source of anthocyanins, which may contribute to the prevention of obesity, which is associated with the reduction of sugars and lipids absorption in the digestive system. Polyphenolic compounds of *A. melanocarpa* can have beneficial effects in reducing blood glucose levels due to inhibition of α-glucosidase and thus preventing the onset of diabetes by controlling postprandial hyperglycemia through the inhibition of α-glucosidase and α-amylase activities. Anthocyanins such as cyanidin 3-rutinoside could potentially inhibit intestinal α-glucosidase, retard the absorption of sugars [115,117,118] and be useful in the prevention and control of diabetes mellitus, as well [119]. Worsztynowicz et al. [115] investigated the effect of black chokeberry extracts on the activity of porcine pancreatic α-amylase and lipase, which are key enzymes in the digestive system. An in vitro study demonstrated that methanolic, water and acetic chokeberry extracts caused inhibition of α-amylase and lipase. The methanolic and acetic extracts exhibited higher inhibitory activities. The most effective inhibitor of pancreatic α-amylase was chlorogenic acid. These findings seem to indicate the use of chokeberry contributing to its anti-obesity activities.

*Aronia* extracts decrease risk factors related to insulin resistance by modulating multiple pathways associated with insulin signaling, adipogenesis and inflammation [116,120]. *Aronia melanocarpa* anthocyanins can normalize the carbohydrate metabolism in diabetic patients and in streptozotocin-diabetic rats [119]. Clinical evidence showed that polyphenol-rich natural products modulate the carbohydrate metabolism by various mechanisms such as restoring beta-cells’ integrity and physiology and enhancing insulin releasing activity. Chokeberries, rich in polyphenols, may decrease the insulin response and therefore be a natural alternative for diabetes treatment [121]. For an improvement of glucose metabolism, Simoneov et al. [117] recommended daily consumption of 200 mL *Aronia* juice for at least three month.

A diet rich in berries is believed to play a distinct role not only in the treatment, but also in the prevention of metabolic diseases. Vlachojannis et al. [122] studied the minimum recommended anthocyanin doses of chokeberry anthocyanins extract for the treatment of metabolic syndrome disorders. They investigated products containing chokeberry due to their usefulness as a functional food. The minimum anthocyanin dose for the treatment of metabolic syndrome disorders was estimated as 110 mg per day. 

### 4.3. Cardioprotective Effect of Chokeberry Fruit

It is well known that chronic inflammatory disorders can lead to cardiovascular diseases characterized by elevated blood pressure, a high level of serum triglycerides and a low level of HDL cholesterol in plasma. In this way, chokeberry fruit derivatives can also have a beneficial effect on several mentioned risk factors for cardiovascular diseases [123,124,125,126]. The fruits are beneficial due to multiple mechanism of action-influence on lipid metabolism, peroxidation, process of inflammation, coagulation and oxidation, as well. 

They effectively influence the lipid metabolism, as has been documented in numerous animal and human studies [123,127,128]. Kim et al. [125] found that *Aronia* extract decreased the expression of genes for cholesterol synthesis, uptake and efflux in a dose-dependent manner in humans. These genes included sterol regulatory element-binding protein 2, scavenger receptor class B Type 1 and ATP-binding cassette transporter A1. *Aronia* extract showed a decreasing of the expression of genes involved in lipid metabolism and lipoprotein assembly, which include fatty acid synthase and acyl-CoA oxidase. There was a significant increase in the levels of LDL receptors and cellular LDL uptake, meaning that cholesterol was taken into the cell, degraded and used in processes like membrane synthesis, steroid production or bile production.

Kardum et al. [128] examined the anthropometric parameters of twenty-nine women (aged 25–49) in relation to markers of lipid peroxidation, before and after 12 weeks of regular chokeberry juice consumption. They found a positive correlation between the regular consumption and marker of lipid peroxidation, further age, body mass index, waist circumference and body fat percent. In the study of Skoczyñska et al. [123], the positive effect of chokeberry juice consumption on lipid parameters was shown: the reduction of the total cholesterol level, LDL cholesterol and triglycerides and increased HDL cholesterol in men with mild hypercholesterolemia without pharmacological treatment. Furthermore, Kowalczyk et al. [124] observed the influence of *Aronia* anthocyanins extract from berries on selected parameters of oxidative stress in young men with hypercholesterolemia that have taken extracted anthocyanins of 240 mg daily for 30 days. Results showed that *Aronia* anthocyanins extract administration caused an increase of glutathione peroxidase and catalase activities in red blood cells. Anthocyanins from *Aronia melanocarpa* can be considered as potent inhibitors of LDL oxidation as a key mechanism of atherosclerosis. It seems that anthocyanins have influenced the blood pressure, endothelin, lipid peroxidation, serum lipids and oxidative status [20] of patients with metabolic syndrome that were treated with *Aronia* extract (3 × 100 mg/day) for two months. After the therapy period, there was a significant decrease of systolic blood pressure, LDL cholesterol, total cholesterol, triglycerides and enzyme catalase and a significant increase of enzyme.

Polyphenolic extract of *A. melanocarpa* berries presented strong anticoagulant properties, prolonged blood clotting times (APTT-activated partial thromboplastin time, PT-prolonged prothrombin time and TT-thrombin time), as well as decreasing the maximal velocity of fibrin polymerization in human plasma. The obtained results clearly indicate the in vitro anticoagulant properties of the berry extracts used [129].

Firstly, the antithrombotic effect is given by inhibiting platelet aggregation. The experiments of Olas et al. [130] showed that the extract from black chokeberries reduces in vitro some steps of platelet activation (platelets adhesion to collagen and platelet aggregation) and the production of reactive oxygen species (ROS) in resting blood platelets and platelets activated by a strong physiological agonist, thrombin. Berries of *A. melanocarpa* have been supposed to prevent thrombosis in pathological states where plasma procoagulant activity and oxidative stress are observed, in hyperhomocysteinemia. In the study of Malinowska et al. [95], the influence of *Aronia* extract on the clot formation (with human plasma and purified fibrinogen) and the fibrin lysis during the model of hyperhomocysteinemia was investigated. The in vitro study presented the modifications of human plasma total proteins and the oxidative stress in plasma after *Aronia* extract application. Ryszawa et al. [131] studied the effects of polyphenol-rich extracts of black chokeberries on platelet function in vitro and in vivo in subjects with significant cardiovascular risk factors, such as hypertension, hypercholesterolemia, smoking and diabetes mellitus. Results in vivo demonstrated that the extract caused a decrease in superoxide production only in patients with cardiovascular risk factors, whereas it was not observed in the group without risk factors for arteriosclerosis. The extract exerted anti-aggregatory effects on platelets in both studied groups. The antioxidant properties of the black chokeberry extract on oxidative/nitrative stress in human blood platelets were studied in vitro. The extract from *A. melanocarpa* significantly inhibited platelet protein carbonylation and thiol oxidation. Especially anthocyanidins, phenolic acids and quercetin glycosides have protective effects against peroxynitrite-induced nitrative damage of plasma fibrinogen and, therefore, may contribute to the prevention of peroxynitrite-related cardiovascular diseases [130]. Furthermore, Bijak et al. [129] estimated the effect of *Aronia* berries’ extract against nitrative and oxidative damage induced by peroxynitrite. The extract significantly inhibited both the formation of the high molecular weight protein aggregates and nitration of the fibrinogen molecule.

*Aronia* fruits significantly inhibited the inflammatory processes in vessels, [17] and protected against atherogenic changes in aorta and coronary arteries, as was documented in the study of Daskalova et al. [132].

Chokeberry fruit extract has also a positive effect on blood pressure and is recommended as a nutritional supplement in the management of arterial hypertension. First, the mechanism of protection is the inhibition of angiotensin I-converting enzyme (ACE). Sikora et al. [120] analyzed the effects of two-month supplementation with chokeberry preparation on the activity of angiotensin I-converting enzyme (ACE) in patients with metabolic syndrome. Results showed a decrease in ACE activity after one (to 25%) and two months (to 30%) of the experiment. They documented significant positive correlations between the ACE activity and the systolic and diastolic blood pressure together with CRP. Bell et al. in two studies [133,134] examined the potential coronary vasoactive and vasoprotective properties of anthocyanin-enriched extract prepared from chokeberry. Chokeberry produced dose- and endothelium-dependent vasorelaxation. Vasorelaxation and its endothelium contribution were least for chlorogenic acid, further caffeic and ferulic acids and greatest for coumaric acid. Thus, anthocyanin-enhanced extracts produce endothelium-dependent relaxation in porcine coronary arteries.

Secondly, the polyphenolic fractions of berries (especially anthocyanins) efficiently decrease oxidative stress. The findings of Kim et al. [135] indicate that *A. melanocarpa* juice is a potent stimulator of the endothelial formation of NO in coronary arteries that involves the phosphorylation of eNOS via the redox-sensitive activation of the Src/PI3-kinase/Akt pathway mostly by conjugated cyanidins and chlorogenic acids. Ciociou et al. [136] estimated the influence of polyphenolic compounds in chokeberries on the parameters of oxidative stress in the model of arterial hypertension in a group of rats without adding extract and with the extract. The serous activity of glutathione-peroxidase has significantly lower values in the hypertensive group as compared to the group protected by polyphenols. The results revealed the normalization of the reduced glutathione concentration, as well as a considerable reduction in the malondialdehyde serum concentration in the group protected by polyphenols. Substances responsible for the mentioned activity are chlorogenic acid, kuromanin, rutin, hyperoside and quercetin. In vitro studies also demonstrated that the *A. melanocarpa* fruit extract inhibits 7β-hydroxycholesterol-induced apoptosis of endothelial cells [103].

### 4.4. Anticancer Effect of Chokeberry Fruit

There is epidemiology evidence indicating that a diet with a high consumption of fruits rich in antioxidants reduces the risk of certain cancer types. Therefore, some dietary antioxidants could be a good prevention of cancer incidence. Results of in vitro experiments proved that *Aronia* fruit has been successfully used as a dietary supplement in some cases of cancer [67]. Moreover, several mechanisms of action have been identified for a chemopreventive effect of polyphenolic extract of black chokeberry fruit (prevention of oxidation, reduction of oxidative stress, induction of detoxication enzymes, induction of cell cycle arrest apoptosis, regulation of the host immune system, anti-inflammatory activity and changes in cellular signaling) [137] that can multiply their utilization in comparison to other berry crops.

The studies of in vitro experiments proved the efficiency of *Aronia melanocarpa* on the growth of human breast, leukemia, colon and cervical tumor cell lines.

Stanisavljević et al. [138] suggested that although a large proportion of chokeberry phenolics undergoes transformation during digestion, they are still potent as antioxidant and antiproliferative agents. The effect of the digested juice on Caco-2 cells proliferation was studied, and the reduction of the proliferative rate by approximately 25% was determined.

Ga̧siorowski et al. [139] established that anthocyanins extract isolated from fruits of *Aronia melanocarpa* inhibited the mutagenic activity of benzo(a)pyrene and 2-amino fluorene in the Ames test. They restrain the generation and release of superoxide radicals by human granulocytes, as well. The results suggest that the antimutagenic influence of anthocyanins is exerted mainly by their free radical scavenging action, as well as by the inhibition of enzymes activating promutagens and converting mutagens to the DNA-reacting derivatives.

Black chokeberry extract and other products may reduce the oxidative stress in breast cancer patients before and after surgery and during various phases of oncology treatment [140]. In a model system in vitro [94], the commercial extract from berries of *A. melanocarpa*, due to the antioxidant action, significantly reduced the oxidative/nitrative stress in platelets from patients with invasive breast cancer after the surgery, as an increase of biomarkers of oxidative stress, such as a decrease of glutathione in platelets from patients with breast cancer, was observed.

The aim of Sharif et al.’s [141] study was to determine the anticancer effect of a polyphenol-rich *Aronia melanocarpa* juice, containing 7.15 g/L of polyphenols, in the acute lymphoblastic leukemia Jurkat cell line. The results of the experiment showed that the juice inhibited cell proliferation, which was associated with cell cycle arrest in the G (2)/M phase, and caused the induction of apoptosis. In a study performed by Sueiro et al. [78], black chokeberry fruits were extracted with aqueous acetone. The extract, some fractions and subfractions were screened in a murine leukemia cell assay and a human DNA catalytic topoisomerase II assay in order to gauge the cancer chemopreventive potential of each genotype. The results of the experiment showed that the anthocyanins extract of black chokeberry inhibited the generation and release of superoxide radicals by human granulocytes. Some of the subfractions extracted from wild and cultivated chokeberry showed >90% of inhibitory activity to leukemia cells at a concentration of 25 and 50 μg/mL. 

Malik et al. [142] revealed that 24-h exposure of 50 μg monomeric anthocyanin/mL of *Aronia* extract to human colon cancer cells resulted in 60% growth inhibition. The treated cells showed a blockage at G1/G0 and G2/M phases of the cell cycle. In the study of Zhao et al. [143], chokeberry anthocyanin-rich extract was also investigated for its potential chemopreventive activity against colon cancer. The growth of colon cancer-derived and non-tumorigenic colonic cells exposed to the extract (10–75 μg of monomeric anthocyanin/mL) was monitored. Colon cancer cell growth was inhibited by 50% after 48 h of exposure to 25 μg/mL extract. 

It is also interesting to compare the inhibition activity of different polyphenolic compounds present in black chokeberry fruit.

Rugină et al. [98] studied polyphenolic fractions of *Aronia melanocarpa* cultivars Viking and Aron, and of *Aronia prunifolia* in relationship with their anticancer activity. Anthocyanin and non-anthocyanin compounds were extracted. The results of the experiment showed that cyanidin glycosides significantly inhibited HeLa human cervical tumor cell proliferation and increased the generation of reactive oxygen species after 48 h of the treatment. Anticancer activity of the extract was dependent on the extraction method. The cancer cell growth inhibition activity of the 50% ethanol extract on the HeLa cell line was significantly higher than that of hot water and of 50% methanol extracts [70]. Summary of protective properties of black chokeberry fruit in relation to chronic diseases are summarized in Table 1.

Since a high intake of fruits is inversely related to the incidence of several degenerative and chronic diseases, the importance of a balanced diet in relation to human health has received increased consumer attention worldwide [144]. Berry crops, especially lesser known species, are effective in the prevention of cardiovascular, cancer and metabolic disorders, as well [145].

## 5. Conclusions

Lesser known fruit species such as black chokeberry, *Aronia melanocarpa*, are coming into the focus of researchers and also the public, particularly due to their great antioxidative activity. They represent a very valuable fruit with a high level of polyphenolic compounds, especially anthocyanins (cyanidin-3-galactoside and cyanidin-3-arabinoside) and procyanidins ((−)-epicatechin units). The content of polyphenols and their exact composition are quite variable and dependent on the particular cultivar, level of fruit ripeness, the growing locality and climatic conditions. Although the bioavailability of polyphenols is poor, their biotransformation can cause the metabolic activation of health benefits. 

Taking into account all of the facts, by the selection of optimal cultivars and growing conditions, polyphenols of *A. melanocarpa* represent a good choice for health-promoting activities including immunomodulatory, antibacterial, hepatoprotective, gastroprotective, cardioprotective, antidiabetic, anti-inflammatory and anticancer benefits.

## Figures and Tables

**Table 1:** Summary of the protective effects of black chokeberry fruit in relation to chronic diseases.

Effect	Mechanism of Action	Study Model	Sources
Anti-inflammatory	Inhibition of release IL-6,IL-8,IL-10 TNF-α Activation NF-κB, cytokines, PGE_2_	Human monocytes, mouse macrophage cells, murine splenocytes	[102,104,105]
	Inhibition on nitric oxide production	Mouse macrophage	[102]
Antidiabetic	Reduction of gastric mucosal demage	Wister rat model	[22]
	Inhibition of α-glucosidase, lipase and amylase	In vitro model studies	[115,117]
	Modulation of multiple pathways associated with insulin signaling	Rats on fructose-rich diet, in vivo, in vitro studies	[118,119]
	Restoring of beta cells integrity	In vivo models	[116,120,121]
Cardioprotective	Lipid metabolism–decrease of cholesterol synthesis	Caco-2-cells	[125]
	-Inhibition of LDL oxidation, lipid peroxidation	Patients with metabolic syndrome	[20]
	-Increase activity of glutathione peroxidase and catalase, reduction of LDL cholesterol -Inhibition of platelet aggregation, protein carbonylation and thiol oxidation	Mens with hypercholesterolemia	[128]
	Anticoagulant, antithrombotic -Decrease in fibrin polymerization	Human plasma	[23,25,123]
	-Inhibition of platelet aggregation, protein carbonylation and thiol oxidation	Human plasma, purified fibrinogen	[95,120,129,130,131]
	Decrease blood pressure		
	-inhibition of angiotensis I-converting enzyme ACE	Patients with metabolic syndrome	[135]
	Protection of coronary arteries		
	Coronary vasoactive, vasoprotective effect	Caco-2-cells	
	Decrease oxidative stress—stimulation of endothelial formation of NO	Porcine coronary arteries	
Anticancer	Inhibition of oxidation	Caco-2-cells line	[138]
	Reduction of oxidative stress	Human granulocytes, HeLa cervical tumor line, murine leukemia cells, brest cancer patients	[94,98,130,139,141]
	Induction of apoptosis	Lymphoblastic leukemia Jurkat cell line	[141]
	Blockage at G1/G0 and G2/M phases of cell cycle	Human colon cancer	[142]
